# CHD4/NuRD complex regulates complement gene expression and correlates with CD8 T cell infiltration in human hepatocellular carcinoma

**DOI:** 10.1186/s13148-020-00827-3

**Published:** 2020-02-18

**Authors:** Simin Shao, Haowei Cao, Zhongkun Wang, Dongmei Zhou, Chaoshen Wu, Shu Wang, Dian Xia, Daoyong Zhang

**Affiliations:** 1 Jiangsu Key Laboratory of Brain Disease and Bioinformation, Research Center for Biochemistry and Molecular Biology, Xuzhou Medical University, Xuzhou, China; 2 Department of Endocrinology, Affiliated Hospital of Xuzhou Medical University, Xuzhou, China

**Keywords:** NuRD, Hepatocellular carcinoma, Immune infiltration, Complement genes

## Abstract

**Backgrounds:**

The NuRD (Nucleosome Remodeling and Deacetylation) complex is a repressive complex in gene transcription by modulating chromatin accessibility of target genes to transcription factors and RNA polymerase II. Although individual subunits of the complex have been implicated in many other cancer types, the complex’s role in human hepatocellular carcinoma (HCC) is not fully understood. More importantly, the NuRD complex has not yet been investigated as a whole in cancers.

**Methods:**

We analyzed the expression of the NuRD complex in HCC and evaluated the prognostic value of NuRD complex expression in HCC using the RNA-seq data obtained from the TCGA project. We examined the effect of CHD4 knockdown on HCC cell proliferation, apoptosis, migration, invasion, epithelial-mesenchymal transition, colony-forming ability, and on complement gene expression. We also performed bioinformatic analyses to investigate the correlation between the NuRD complex expression and immune infiltration.

**Results:**

We found that nine subunits, out of 14 subunits of the NuRD complex examined, were significantly overexpressed in HCC, and their expression levels were positively correlated with cancer progression. More importantly, our data also demonstrated that these subunits tended to be overexpressed as a whole in HCC. Subsequent studies demonstrated that knockdown of CHD4 in HCC cells inhibits cell proliferation, migration, invasion, and colony-forming ability and promotes apoptosis of HCC cells, indicating that the CHD4/NuRD complex plays oncogenic roles in HCC. Further analysis revealed that the CHD4/NuRD complex regulates complement gene expression in HCC. Intriguingly, we found that the CHD4/NuRD complex expression was inversely correlated with CD8 T cell infiltration in HCC.

**Conclusions:**

Our data demonstrate that the CHD4/NuRD complex plays an oncogenic role in human HCC and regulates complement gene expression in HCC cells. The results of inverse correlation between the CHD4/NuRD complex and CD8 T cell and DC cell infiltration in HCC suggest that the CHD4/NuRD complex not only plays direct regulatory roles in HCC cells, but also has an impact on the immune microenvironment of HCC.

## Introduction

The NuRD (Nucleosome Remodeling and Deacetylation) complex is one of four major ATP-dependent chromatin remodeling complexes, and it functions as a transcriptional repressor in gene regulation by coordinating ATP-dependent chromatin remodeling helicase and histone deacetylase activity to modulate chromatin accessibility of target genes to transcription factors and RNA polymerase II [1–4]. The NuRD complex is composed of multiple subunits, including ATP-dependent chromatin remodeling helicase CHD3/CHD4 (also known as Mi-2α/Mi-2β), histone deacetylase HDAC1/HDAC2, RBBP4/RBBP7 (also known as RbAp48/46), GATAD2A/GATAD2B, MTA1/MTA2/MTA3, the mCpG-binding domain protein MBD2/MBD3, and the histone demethylase KDM1A [2–7]. The NuRD complex is ubiquitously expressed in nearly all the tissues, and it is highly conserved from plants to animals. The NuRD complex has been reported to play a crucial role in a variety of cellular processes, including stem cell biology, cell cycle regulation, genome integrity maintenance, DNA damage repair, and development of the immune system [8–15].

Many subunits of the NuRD complex have been implicated in oncogenesis and cancer progression by regulating the related transcriptional events or through its non-transcriptional roles in DNA damage repair, chromatin assembly, and the maintenance of genomic integrity. MTA1 is the best-studied subunit of the NuRD complex in the context of tumor biology. MTA1 has been shown to be overexpressed in various cancers, including breast cancer, pancreatic cancer, and ovarian cancer, and its overexpression correlates with cancer progression and poor outcome in many cancer types [16]. Many lines of evidence lead to a conclusion that oncogenic transcription factors recruit the NuRD complex to repress downstream target genes that are integral to tumorigenesis. For instance, the NuRD complex subunit MTA3 has been shown to directly interact with BCL-6, an oncogene that plays a crucial role in DLBCL (diffuse large B cell lymphoma) [17, 18]. The MBD3 subunit was shown to directly interact with JUN, an oncogene that plays important roles in many malignancies [19].

Although many subunits of the NuRD complex have been implicated in various types of cancer, its role in hepatocellular carcinoma is not very clear. Moreover, the NuRD complex has not yet been studied as a whole in the context of tumor biology so far. In the present study, we evaluate the expression of the NuRD complex as a whole in hepatocellular carcinoma and investigate the pathways regulated by the NuRD complex. We also demonstrate that the expression of the NuRD complex inversely correlates with CD8+ T cell and DC cell infiltration in hepatocellular carcinoma which are key immune cell types in tumor immunotherapy.

## Material and methods

### Gene expression and clinical phenotype data source

The data for mRNA expressions (mRNA SeqV2) and clinical phenotypes of human liver hepatocellular carcinoma were obtained from The Cancer Genome Atlas (TCGA) project. The detailed clinicopathological data for each sample, including the pathologic stage and histologic grade information, can be accessed via the NIH GDC Data Portal. The gene expression profile was measured in 371 primary hepatocellular carcinoma (HCC) cases and 50 normal liver tissue samples using the Illumina HiSeq 2000 RNA Sequencing platform by the University of North Carolina TCGA genome characterization center.

### Cell culture and gene silencing

Human hepatocellular carcinoma cell lines were originally obtained from the American Type Culture Collection (ATCC). HuH-7 cells were grown in Dulbecco’s modified Eagle’s medium (Invitrogen) containing 10% fetal calf serum. Hep3B cells were grown in MEM supplemented with 10% fetal calf serum, Glutamax, non-essential amino acids, and sodium pyruvate. SNU-387 cells were maintained in RPMI 1640 Medium containing 10% fetal calf serum, Glutamax, and sodium pyruvate. The cell culture was maintained at 37 °C in a humid atmosphere containing 5% CO_2_.

The shRNAs targeting CHD4 (CHD4-sh1: GCCTAAACCCAAGAAAGTAGC; CHD4-sh2: GCGGCAGTTCTTTGTGAAATG) and a negative control shRNA with scrambled sequence (shControl: TTCTCCGAACGTGTCACGT) were constructed into pLV3 lentivirus vector (GenePharma, China). Lentiviruses were produced by the transfection of 293 T cells with plasmids using packaging Mix (GenePharma, China). To knock down CHD4 expression, hepatocellular carcinoma cells were infected with the lentiviruses and selected with puromycin for 2 weeks.

### RNA extraction, reverse transcription, and quantitative real-time PCR

Total RNA was extracted from cells with TRIzol (Invitrogen) according to the manufacturer’s instruction. cDNA was generated by reverse transcription using HiScript III RT SuperMix for qPCR (+g DNA wiper) purchased from Vazyme. Quantitative real-time PCR was performed on an ABI-7500 using TB Green™ Premix Ex Taq™ (Tli RNaseH Plus) (TAKARA). Actin was used for normalization of qRT-PCR data.

### Western blotting, immunofluorescence, and antibodies

Cells were lysed in RIPA lysis buffer (50 mM Tris-HCl, pH 8.0, 150 mM NaCl, 0.5% sodium deoxycholate, 1% NP-40, and 0.1% SDS) containing protease inhibitor cocktail (Roche). Protein lysates were subjected to SDS-PAGE, transferred to PVDF membrane (Millipore), and probed with appropriate primary antibodies and HRP-conjugated secondary antibodies by ECL reagent (GE Healthcare).

Cells seeded and grown on glass coverslips in 24-well plates were fixed in 4% formaldehyde solution and permeabilized with PBS containing 0.2% Triton X-100. Cells were blocked in PBS containing 5% bovine serum albumin for 1 h and incubated with primary antibody at room temperature for 1 h, followed by incubation with fluorescent dye-conjugated secondary antibodies for 1 h, and then stained with DAPI. All images were captured with a Leica microscope.

The following antibodies were used in western blotting and immunofluorescence: anti-Vimentin (Proteintech, 10366-1-AP), anti-β-Catenin (Proteintech, 51067-2-AP), anti-CHD4 (Proteintech, 14173-1-AP), and anti-β-Tubulin (Proteintech, 66240-1-Ig). Fluorescent dye-conjugated secondary antibodies used were Alexa Fluor 647-conjugated goat anti-rabbit (Beyotime, A0468).

### CCK-8 assay and clonogenic assay

Cell viability was measured using Cell Counting Kit-8 (Beyotime, C0038) following the manufacturer’s protocol. Cells were seeded at a density of 3 × 10^3^ cells per well in 96-well plates with four replicates. One hundred microliters of serum-free cell culture medium containing 10 μl of WST-8 reagent was added at desired time points and incubated in cell culture incubator for 2 h. Optical absorbance of each well at 450 nm was measured with a microplate reader. For clonogenic assay, cells were seeded in 6-well plate at 1000 cells/well followed by incubation in cell culture incubator for 14 days. Cells were then stained with crystal violet staining solution (Beyotime, C0121) and counted manually. Three independent experiments were performed for statistical analysis.

### Wound healing assay and transwell cell invasion assay

For wound-healing assay, cells were seeded and grown in six-well plates. Cells were first serum starved overnight, and then cell layers were wounded using a sterile 1-ml pipette tip. The detached cells were washed away with PBS, and then, the cells were cultured in medium supplemented with 1% FBS. Gap areas were photographed with a light microscope at desired time points.

Twenty-four-well inserts transwell chambers (8.0 mm, Corning) were used for transwell in vitro cell invasion assays. 2 × 10^5^ cells were seeded into the top chamber coated with Matrigel (BD Biosciences). Complete medium was added to the bottom wells to stimulate cell invasion. After incubation for 24–48 h in cell culture incubator, cells that did not penetrate through the membrane were removed with a cotton swab. Cells adhered to the lower surface of the membrane were stained with 0.1% crystal violet and cells were counted manually.

### Statistical analyses

Receiver operating characteristic (ROC) analysis was carried out to ascertain the diagnostic performance for each of the NuRD complex subunits, and the optimal cutoff value for the expression of each of the NuRD complex subunits was determined based on Youden index. The expression levels of each of the NuRD complex subunits were categorized into high and low expression groups based on the cutoff value. Log-rank test was used to assess the difference between the survival curves. Gene Set Enrichment Analysis (GSEA) was performed using the Bioconductor package clusterProfiler based on KEGG pathways (minimal set size 20, maximal set size 500) [20].

## Results

### The NuRD complex was aberrantly overexpressed in human hepatocellular carcinoma

**Fig. 1:**
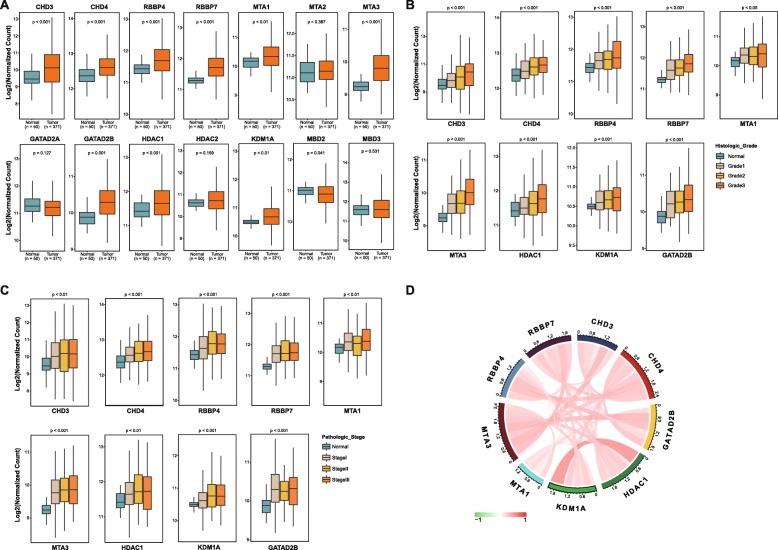
Expression of the NuRD complex was upregulated in human hepatocellular carcinoma. **a** Expression of 14 subunits of the NuRD complex was examined in human hepatocellular carcinoma samples (*n* = 371) and normal liver tissue samples (*n* = 50). **b**, **c** The 9 subunits overexpressed in HCC are positively correlated with cancer progression. Cases lacking histologic grade information or pathologic stage information were not included in the corresponding analysis. **d** Pearson correlation analyses were carried out to assess the correlation among the NuRD complex subunits

To investigate the role of the NuRD complex in human hepatocellular carcinoma (HCC), we first analyzed the expression levels of each of the NuRD complex subunits in HCC and in normal liver tissue samples. We found that out of 14 subunits analyzed, the expression levels of 9 subunits were significantly upregulated in HCC samples compared to that in normal liver tissue samples (Fig. 1a). We further analyzed the expression of these subunits in HCC samples of different pathologic stages and histologic grades. Cases lacking histologic grade information or pathologic stage information were excluded from the corresponding analyses. The results showed that the expression levels of these subunits were positively correlated with cancer progression (Fig. 1b, c).

Although our data revealed that most subunits of the NuRD complex were upregulated in HCC, whether these subunits were co-overexpressed in the same cohort of samples is not clear. Thus, we performed Pearson correlation analysis to evaluate the correlation between these subunits in HCC and found that the expression of these subunits was positively inter-correlated in HCC (Fig. 1d). The result indicates that these subunits overexpressed in HCC tend to be overexpressed in the same cohort of HCC samples, supporting a hypothesis that the NuRD complex was overexpressed in HCC as a whole.

### High NuRD expression correlates with poor overall survival in patients with HCC

**Fig. 2:**
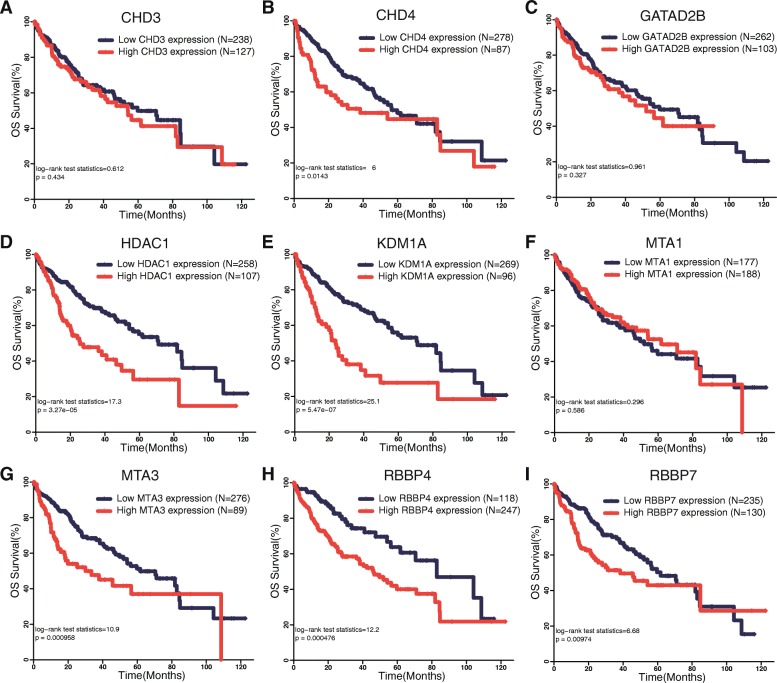
High expression of the NuRD complex is associated with poor prognosis of HCC patients. The expression levels of each of the nine NuRD complex subunits overexpressed in HCC were categorized into high expression group and low expression group using the optimal cutoff value determined based on Youden index. Log-rank test was performed to assess the difference between the survival curves. The number of cases analyzed in each group is indicated

Next, we continued to assess the prognostic value of the expression of the NuRD complex for human hepatocellular carcinoma. The expression levels of each of the nine NuRD complex subunits which were upregulated in HCC were categorized into high expression group and low expression group using the optimal cutoff value determined based on Youden index. Log-rank test was performed to assess the difference between the survival curves. The results showed that among the nine subunits analyzed, the expression levels of six subunits were associated with significantly worse overall survival probability in patients with HCC (Fig. 2).

Although the NuRD complex is highly conserved from plants to animals and is ubiquitously expressed in nearly all the tissues, the composition of the NuRD in different tissues and under different pathologic conditions is quite different. The specific combinatorial assembly of the complex determines the cell type-specific functions. Our data suggest that the NuRD complex composed of CHD4, HDAC1, KDM1A, RBBP4, RBBP7, and MTA3 along with other core subunits may play oncogenic roles in human HCC.

### Knockdown of CHD4 inhibits proliferation and promotes apoptosis of HCC cells

**Fig. 3:**
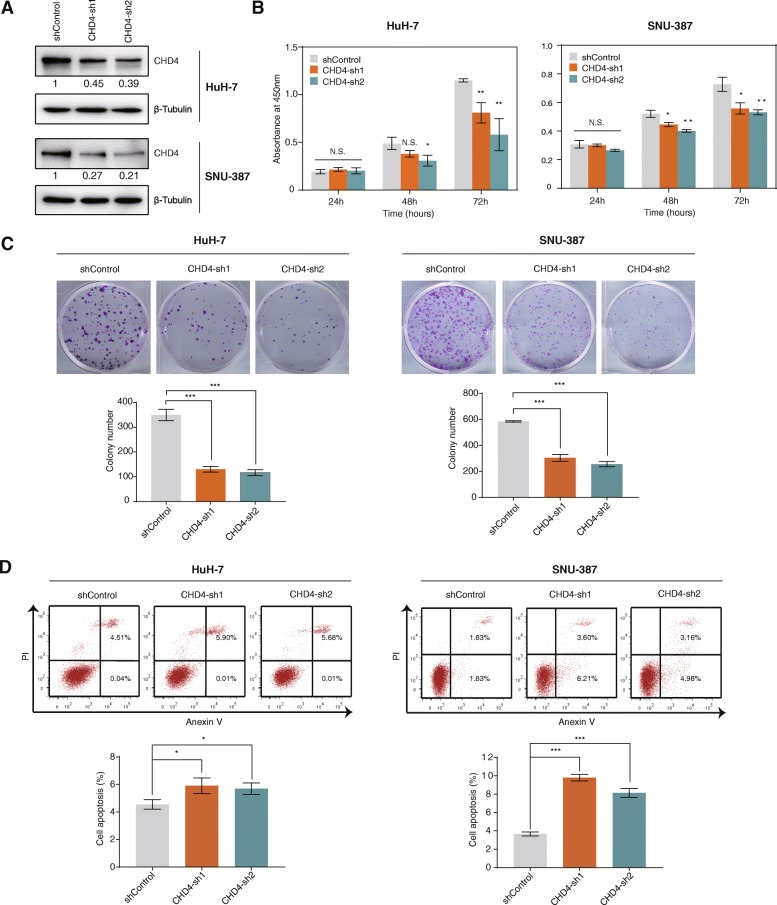
Knockdown of CHD4 inhibits proliferation and colony-forming ability and increases apoptosis of HCC cells. **a** The expression levels of CHD4 were detected in CHD4 knockdown cell lines and negative control cell lines using western blotting analyses. β-Tubulin was used as loading control. **b** CCK-8 assay was performed to assess the effect of CHD4 knockdown on HCC cell proliferation. **c** Clonogenic assay was performed to examine the effect of CHD4 knockdown on colony-forming ability of HCC cells. **d** The effect of CHD4 knockdown on apoptosis of HCC cells was examined by flow cytometry analysis. *p < 0.05, **p < 0.01, ***p < 0.001, unpaired *t* test

To further investigate the role that the NuRD complex plays in HCC, we knocked down expression of CHD4 subunit in HCC cells and carried out CCK-8 assay to study the complex’s role in HCC cell proliferation (Fig. 3a). We chose CHD4 to knock down because CHD4 is the ATP-dependent helicase subunit of the NuRD complex. Knockdown of the CHD4 subunit would impair the ability of the CHD4/NuRD complex in remodeling chromatin and in regulating expression of its target genes. Results showed that knockdown of CHD4 significantly inhibited cell proliferation of both HuH-7 cells. Similar effect was also observed in another HCC cell line, SNU-387 cells (Fig. 3b).

Next, we carried out clonogenic assay to assess the role of the complex in regulating the colony-forming ability of the HCC cells. As seen in Fig. 3c, CHD4 knockdown dramatically reduced the colony-forming ability of HCC cells. In addition, CHD4 knockdown significantly increased apoptosis in both HuH-7 cells and SNU-387 cells (Fig. 3d). Taken together, our data suggest that the CHD4/NuRD complex promotes cell proliferation and inhibits apoptosis in HCC.

### CHD4 knockdown inhibits HCC cell migration and invasion

**Fig. 4:**
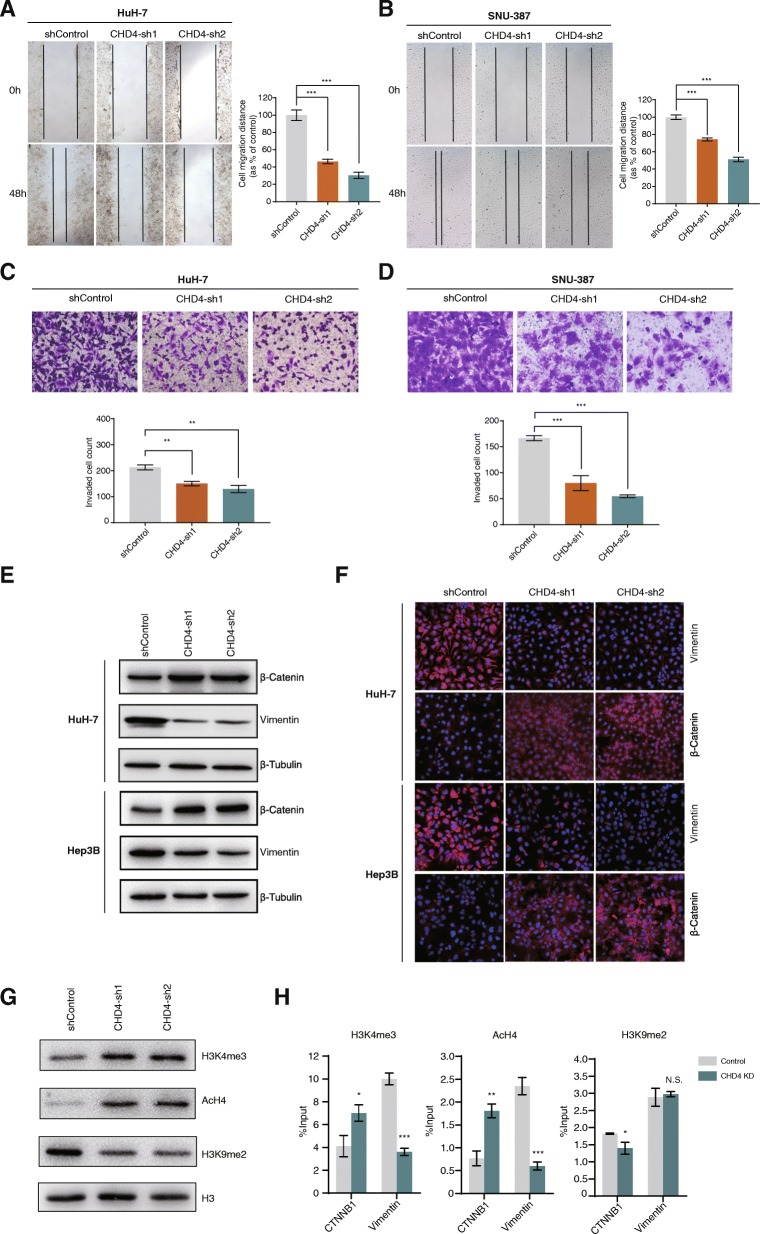
Knockdown of CHD4 inhibits HCC cell migration and invasion. **a**, **b** Wound healing assay was performed to examine the effect of CHD4 knockdown on HCC cell migration. **c**, **d** Transwell invasion assay was performed to examine the effect of CHD4 knockdown on HCC cell invasion. **e**, **f** Expression levels of the EMT marker genes were detected in CHD4 knockdown cell lines and negative control cell lines by western blotting analysis and immunofluorescence staining. **g** Western blotting assay to examine the effects of CHD4 knockdown on histone modifications. **h** ChIP assay to examine the effects of CHD4 knockdown on histone modifications at the promoter of the *CTNNB1* and *Vimentin* genes. *p < 0.05, **p < 0.01, ***p < 0.001, unpaired *t* test

Next, we continued to investigate the role of CHD4 in HCC cell migration and invasion. Wound healing assay was carried out to study the effect of CHD4 knockdown on HCC cell migration. As seen in Fig. 4a, knockdown of CHD4 in HuH-7 cells significantly inhibited cell migration. Similar results were also observed in SNU-387 cells (Fig. 4b). To investigate the role of CHD4 in HCC cell invasion, transwell cell invasion assay was performed. Similar to the wound healing assay results, CHD4 knockdown significantly reduced HCC cell invasion (Fig. 4c, d). These data suggest that CHD4/NuRD may promote cell migration and invasion in HCC. In line with the above results, based on marker gene expression, we also observed mesenchymal-epithelial transition (MET)-like change upon CHD4 knockdown in HCC cells, indicating that the CHD4/NuRD complex may promote epithelial-mesenchymal transition (EMT) in HCC cells (Fig. 4e, f).

Next, we examined the effects of CHD4 knockdown on chromatin modifications globally. As shown in Fig. 4g, we observed elevated levels of total H3K4 methylation and H4 acetylation and reduced levels of total H3K9 methylation upon CHD4 knockdown. Given the fact that methylated H3K4 and acetylated H4 are generally associated with active chromatin while methylated H3K9 is generally associated with inactive chromatin, our data indicates that the CHD4/NuRD complex may suppress transcription globally in hepatocellular carcinoma. We further performed ChIP experiments to examine the effects of CHD4 knockdown on chromatin status at the promoter of the EMT marker genes. As shown in Fig. 4h, we observed elevated levels of H3K4 methylation and H4 acetylation and slightly reduced levels of H3K9 methylation at the promoter of the *CTNNB1* gene upon CHD4 knockdown. At the promoter of the *Vimentin* gene, we observed reduced H3K4 methylation levels and H4 acetylation levels while H3K9 methylation levels remained unchanged. These results are consistent with the changes in protein levels of the two EMT marker genes observed in western blotting assay. The opposite change in chromatin status at the promoter of the *vimentin* gene indicates that the *Vimentin* gene may be an indirect target of the CHD4/NuRD complex.

### The CHD4/NuRD complex suppresses expression of the complement system genes

The above results lead to a conclusion that the CHD4/NuRD complex plays an oncogenic role in HCC. Next, we sought to identify pathways and genes regulated by the CHD4/NuRD complex in human HCC. To this end, we compared the gene expression profiles of the high_expression HCC samples to that of the low_expression HCC samples for each of the six subunits (CHD4, KDM1A, HDAC1, MTA3, RBBP4, RBBP7) of the NuRD complex, which are overexpressed in HCC and show prognostic value, to identify differentially expressed genes using DESeq2 package, an R/Bioconductor package. We further performed Gene Set Enrichment Analysis (GSEA) to identify pathways that were altered in the high_expression group compared to the low_expression group for each of the six subunits.

**Fig. 5:**
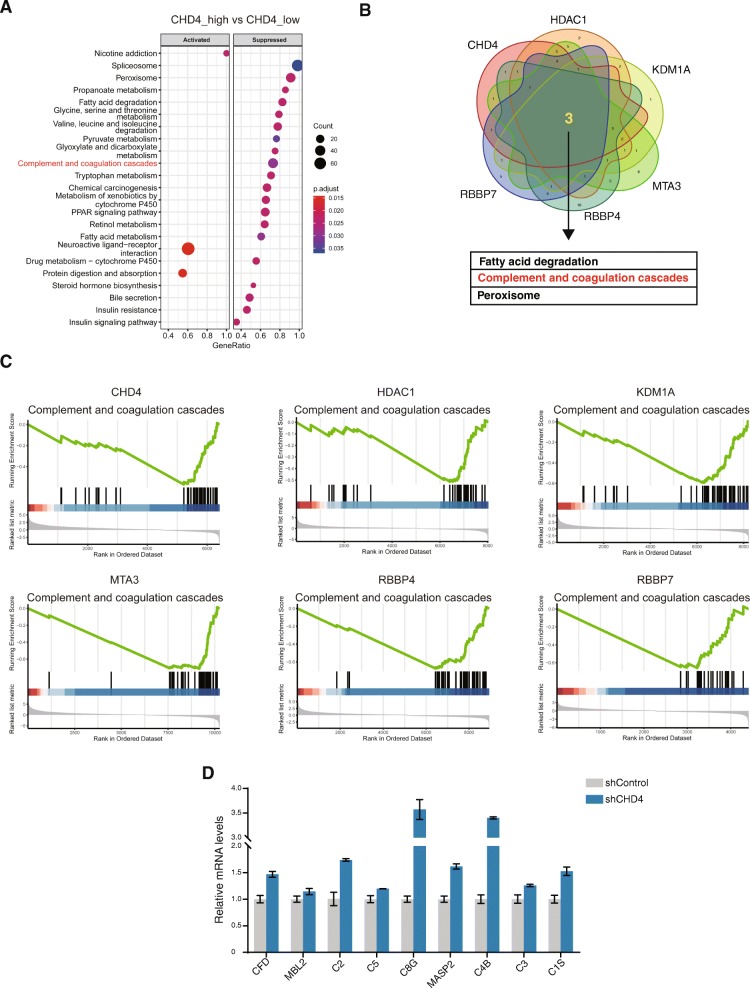
The CHD4/NuRD complex regulates complement gene expression. **a** Gene Set Enrichment Analysis (GSEA) analysis was performed to identify the pathways altered in CHD4 high_expression HCC samples compared to CHD4 low_expression samples. **b** Venn diagram to show the altered pathways common to all of the six subunits of the NuRD complex which are overexpressed and correlated with poor prognosis in HCC. **c** GSEA enrichment score curves to show that the complement and coagulation cascades are downregulated in high_expression HCC samples for all of the six subunits. **d** qRT-PCR analyses were performed to examine the effect of CHD4 knockdown on complement gene expression in HCC cells

As shown in Fig. 5a and Supplementary Figure S1, most altered pathways in the high_expression group compared to the low_expression group were downregulated, consistent with the complex’s repressive role in gene regulation. When we compared the pathways affected in high_expression group among the six subunits, we found that the complement and coagulation cascades, along with the other two pathways, were downregulated in high_expression group for all the six subunits (Fig. 5a–c and Supplementary Figure S1). To investigate this further, we carried out qRT-PCR to verify the effect of CHD4 knockdown on expression of the complement system genes in HCC cells. As shown in Fig. 5d, CHD4 knockdown significantly increased expression of C4B, along with other complement system genes examined in the assay. C4B is a critical component in the complement system since it commences the initial step of the system activation. Our data indicate that the CHD4/NuRD complex suppresses C4B expression in HCC.

### The CHD4/NuRD complex inversely correlates with CD8 T cell infiltration in human hepatocellular carcinoma

**Fig. 6:**
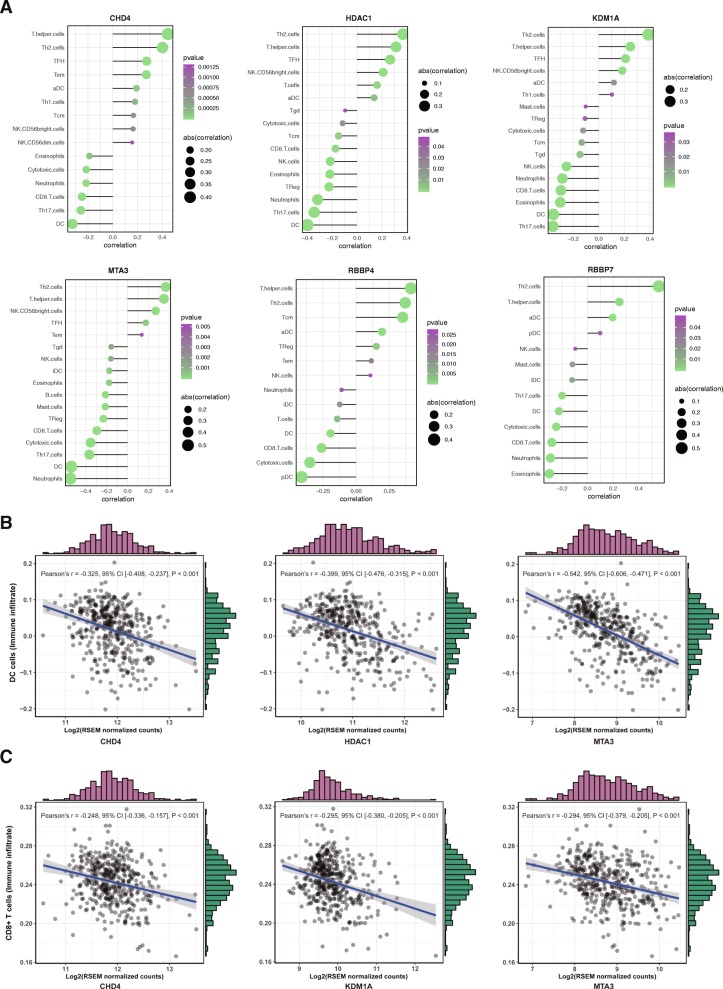
The CHD4/NuRD complex inversely correlates with CD8 T cell infiltration in human hepatocellular carcinoma. The infiltration levels of immune cell types in each of the HCC samples (*n* = 371) were quantified using Single-Sample Gene Set Enrichment Analysis (ssGSEA) in R package gsva. The gene signatures expressed by immune cell populations were applied in ssGSEA for each of the HCC samples. The correlation between the expression of the six subunits and the infiltration levels of the immune cell types in HCC samples were then analyzed and the results are shown

Numerous studies and clinical data have attached great importance to immune infiltration in solid tumors with respect to immune checkpoint immunotherapies. Therefore, we wondered whether the CHD4/NuRD complex had an impact on tumor immune microenvironment in HCC. To this end, we first quantified infiltration levels of immune cell types in each of the HCC samples (*n* = 371) using Single-Sample Gene Set Enrichment Analysis (ssGSEA) in R package gsva. The gene signatures expressed by immune cell populations [21] were applied in ssGSEA for each of the HCC samples. Then, we analyzed the correlation between the expression of the six subunits of the CHD4/NuRD complex and the infiltration levels of the immune cell types in HCC samples. As shown in Fig. 6a, b, the expression of the CHD4/NuRD complex inversely correlates with CD8 T cell and DC cell infiltration in HCC, both are critical for tumor immune surveillance. Our results suggest that in addition to its direct regulatory roles in HCC cells, the CHD4/NuRD complex may also have an impact on tumor immune microenvironment of HCC.

## Discussion

Here, we explored the role of the NuRD complex in HCC. We first examined the expression of the NuRD complex in HCC samples as well as in normal liver tissue samples and found that 9 subunits out of 14 subunits of the NuRD complex examined were significantly upregulated in HCC samples compared to that in normal liver tissue samples. More importantly, their expression was positively inter-correlated, suggesting that these subunits were overexpressed as a whole and function in the context of the NuRD complex, not as an independent individual gene, in the oncogenesis of HCC. Further analysis revealed that higher expression levels of six subunits, i.e., CHD4, HDAC1, KDM1A, MTA3, RBBP4, RBBP7, were associated with more advanced stage, higher grade, and poorer prognosis, proving the importance of the NuRD complex with this specific composition in HCC. Given the fact that CHD4 is the ATP-dependent helicase subunit of the NuRD complex, knockdown of the CHD4 subunit would impair the chromatin-remodeling ability of the CHD4/NuRD complex. Therefore, we performed various experiments to assess the effect of CHD4 knockdown in HCC cells. The results indicate that the CHD4/NuRD complex promotes cell proliferation, migration, invasion, and colony-forming ability and represses apoptosis of HCC cells. Taken together, our data support an oncogenic role of the CHD4/NuRD complex in HCC.

Further mechanistic study revealed that the CHD4/NuRD complex regulated complement gene expression in HCC. C4B expression was dramatically upregulated upon CHD4 knockdown along with other complement genes. C4B is a critical component of the complement system as it commences the initial step of activation the complement system [22]. The involvement of the complement genes in tumorigenesis has been reported in many cancer types. However, the conclusions are contradictory [23], raising the possibility that whether the complement system plays a positive or negative regulatory role in tumorigenesis depends on the cancer type. Since some components of the complement system have been reported to function independent of complement system in cancers [24], another possibility is that these complement genes repressed by the CHD4/NuRD complex, including C4B, may play a regulatory role in cancer progression independent of complement system. Further studies are needed to address these possibilities. Tumor infiltration lymphocytes play essential roles in anti-tumor immune responses. Here, we analyzed the correlation between the CHD4/NuRD complex and immune infiltration in HCC. Results showed that all the six subunits, which are overexpressed in HCC and associated with poor prognosis, were inversely correlated with CD8 cell and DC cell infiltration in HCC, both are critical immune cell types in anti-tumor immune response.

As a major transcription regulator, the NuRD complex is thought to regulate oncogenesis in many aspects. For example, CHD4 has been shown to regulate EpCAM^+^ liver cancer stem cells and chemoresistance in human hepatocellular carcinoma, supporting the hypothesis that the NuRD complex regulates oncogenesis via multiple mechanisms [25]. Since the NuRD complex is a HDAC-containing chromatin remodeling complex and the HDAC activity pivotally contributes to the gene regulation ability of the NuRD complex, targeting HDAC may help treat liver cancer, especially in NuRD-overexpressing liver cancer. Indeed, many HDAC inhibitors have demonstrated antitumor efficacy in clinical studies [26]. Moreover, since our results potentially link the NuRD complex to immune infiltration in hepatocellular carcinoma, targeting the HDAC activity within the NuRD complex may synergize with immune checkpoint inhibitor treatment. In line with this hypothesis, there is evidence showing that HDAC inhibitor treatment indeed enhanced anti-tumor efficacy of checkpoint inhibitors in hepatocellular carcinoma [27]. As a chromatin remodeling enzyme subunit in the NuRD complex, CHD4 is pivotal to the chromatin remodeling activity of the complex as well as its ability in regulating target gene expression. Therefore, targeting CHD4 may paralyze the complex, leading to deregulation of the target genes, including EMT-related genes and the complement pathway genes, and eventually affect tumorigenesis in hepatocellular carcinoma.

In conclusion, our data demonstrate that the CHD4/NuRD complex plays an oncogenic role in human hepatocellular carcinoma. Mechanistic study reveals that the CHD4/NuRD complex regulates complement gene expression, possibly linking the complement system to cancer progression in HCC. Intriguingly, in addition to its direct regulatory roles in HCC cells, our results also showed that the CHD4/NuRD complex inversely correlated with CD8 T cell and DC cell infiltration in HCC, suggesting that the CHD4/NuRD complex not only plays direct regulatory roles in HCC cells, but also has an impact on the immune microenvironment of HCC.

## Supplementary information

**Additional file 1: Table S1.** Primer pairs for real-time PCR. **Table S2.** Primer pairs for ChIP-QPCR. **Figure S1.** The CHD4/NuRD complex regulates complement gene expression. Gene Set Enrichment Analysis (GSEA) analysis was performed to identify the pathways altered in high_expression HCC samples compared to low_expression samples for subunits HDAC1, KDM1A, MTA3, RBBP4 and RBBP7 respectively. **Figure S2.** CHD4 knockdown efficiency was measured in Hep3B cells using western blotting analyses. β-Tubulin was used as loading control.

## Data Availability

The data used and/or analysis during the current study are available from the corresponding author on reasonable request.

## Abbreviations

HCC: Hepatocellular carcinoma
NuRD: Nucleosome Remodeling and Deacetylation
TCGA: The Cancer Genome Atlas
DLBCL: Diffuse large B cell lymphoma
GSEA: Gene Set Enrichment Analysis
