# Supplementary material for: CHD4/NuRD complex regulates complement gene expression and correlates with CD8 T cell infiltration in human hepatocellular carcinoma
Source: Clin Epigenetics. 2020 Feb 18;12:31. doi: 10.1186/s13148-020-00827-3 (PMC7027061; doi:10.1186/s13148-020-00827-3)
Supplement: Supplementary file 1 — Additional file 1: Table S1. Primer pairs for real-time PCR. Table S2. Primer pairs for ChIP-QPCR. Figure S1. The CHD4/NuRD complex regulates complement gene expression. Gene Set Enrichment Analysis (GSEA) analysis was performed to identify the pathways altered in high_expression HCC samples compared to low_expression samples for subunits HDAC1, KDM1A, MTA3, RBBP4 and RBBP7 respectively. Figure S2. CHD4 knockdown efficiency was measured in Hep3B cells using western blotting analyses. β-Tubulin was used as loading control. [file 13148_2020_827_MOESM1_ESM.docx]

**Supplementary table 1: Primer pairs for real-time PCR**

| Genes | Sequences(5’-3’) | |
| --- | --- | --- |
| CFD | Sense | CAGCTGCAAGGGTGACTCC |
|  | Antisense | CAGGACGCTGTCGATCCAG |
| MBL2 | Sense | GTGATGGCACCAAGGGAGAA |
|  | Antisense | TCACCATCCGGACTTTTTCCA |
| C2 | Sense | GGGGACAAGGTCCGCTATC |
|  | Antisense | GAAGTCATAAGAGTAGGGTTGGC |
| C5 | Sense | ATTCGCTTGACCAGTTGGTAG |
|  | Antisense | CCAGATGGGAGATTAAGCACAAA |
| C8G | Sense | CCGACTACCAGAGTTTCGCT |
|  | Antisense | CAGAAGCCGTACTTGGGGAAG |
| MASP2 | Sense | TCCAGGGGAGTATGCCAATGA |
|  | Antisense | TCCAGGTCGAAGTGGGTGAA |
| C4B | Sense | ATGAAGAACACGACCTGCCA |
|  | Antisense | TTCCTCCGACCCTCAAACAG |
| C3 | Sense | TACTACACGCTGATCGGTGC |
|  | Antisense | GCTTTTTACCACCAGCGAGC |
| C1S | Sense | TTTGGCATGGGTTTATGCTGA |
|  | Antisense | GGGTGAAGTAGAGGTGAATCCC |

**Supplementary table 2: Primer pairs for ChIP-QPCR**

| Genes | Sequences(5’-3’) | |
| --- | --- | --- |
| VIM | Sense | CCTGAGGAATCGGCTATGGG |
|  | Antisense | CTTCTACGTGCAGTGCCGA |
| CTNNB1 | Sense | AAGAATACGCTGGCCCTGAA |
|  | Antisense | AACACATAGATGCAGGCGGA |

**Supplementary Figures**


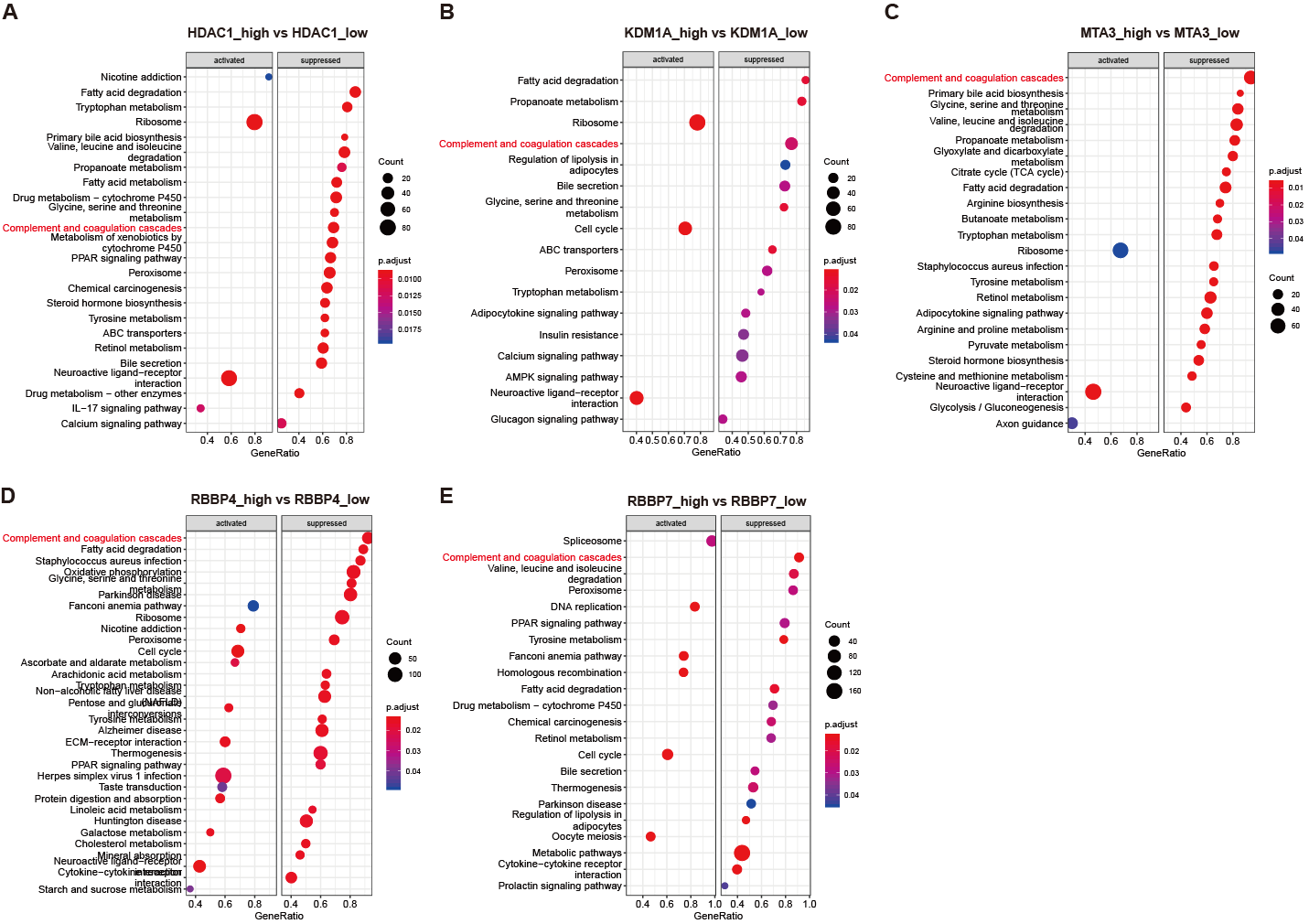


**Supplementary Figure S1.** The CHD4/NuRD complex regulates complement gene expression. Gene Set Enrichment Analysis (GSEA) analysis was performed to identify the pathways altered in high_expression HCC samples compared to low_expression samples for subunits HDAC1, KDM1A, MTA3, RBBP4 and RBBP7 respectively.

**Supplementary Figure S2.** CHD4 knockdown efficiency was measured in Hep3B cells using western blotting analyses. β-Tubulin was used as loading control.
